# Shared Genetic Architecture Among Severe Mental Disorders: A System Biology Approach Based on Protein–Protein Interaction

**DOI:** 10.1002/brb3.70742

**Published:** 2025-09-08

**Authors:** Hernan F. Guillen‐Burgos, Valentina Vanegas, Isabella Gonzalez, Ana M. Caicedo, Valentina Arango, Juan F. Galvez‐Florez, Juan‐Manuel Anaya, Roger S. McIntyre, Wilson Terán

**Affiliations:** ^1^ Universidad Simón Bolivar Center for Clinical and Translational Research Barranquilla Colombia; ^2^ Center for Clinical and Translational Research Bogotá Colombia; ^3^ Pontificia Universidad Javeriana Department of Psychiatry and Mental Health Bogotá Colombia; ^4^ Universidad Del Rosario School of Medicine Bogotá Colombia; ^5^ Pontificia Universidad Javeriana Faculty of Medicine Bogotá Colombia; ^6^ Universidad De La Costa Faculty of Medicine Barranquilla Colombia; ^7^ Department of Psychiatry University of Toronto Toronto Ontario Canada; ^8^ Pontificia Universidad Javeriana Facultad De Ciencias, Departamento de Biología, Biología de Plantas y Sistemas Productivos Bogotá Colombia

**Keywords:** neuroinflammation, protein–protein interaction, shared genetic architecture, severe mental disorders, synaptic plasticity

## Abstract

**Introduction:**

The study explores shared genetic architecture among major psychiatric disorders—major depressive disorder, bipolar disorder, schizophrenia, and post‐traumatic stress disorder—emphasizing their overlapping molecular pathways. Using public datasets, we identified shared genes and examined their functional implications through protein–protein interaction (PPI) networks and gene set enrichment analysis (GSEA).

**Methods:**

Genes associated with each disorder were identified through the NCBI Gene database. Intersection analyses of gene sets were conducted using R to identify overlaps among the four disorders. STRING was used to predict PPI and conduct clustering analyses. Gene set enrichment analysis was performed to explore biological pathways, molecular functions, and cellular components.

**Results:**

We identified 31 intersected genes across all four disorders. PPI analyses demonstrated significant network enrichment, revealing interconnected pathways related to inflammation, neurotransmission, and synaptic plasticity. Functional enrichment highlighted pathways such as cytokines signaling, dopaminergic transmission, and synaptic vesicle cycling. Tissue expression analysis indicated significant involvement of brain regions, including the anterior cingulate cortex and mesolimbic system.

**Conclusion:**

This study underscores the shared genetic underpinnings of severe psychiatric disorders, highlighting common biological processes, such as pro‐inflammatory markers and synaptic signaling. These findings offer a transdiagnostic perspective, potentially informing novel therapeutic strategies for overlapping psychiatric conditions.

## Graphical abstract

This study explores the genetic overlap among PTSD, MDD, BD, and SZ, revealing shared biological pathways and co‐expression networks. Functional enrichment analysis highlights common molecular mechanisms underlying these psychiatric disorders, providing insights into their comorbidity and potential avenues for targeted therapeutic strategies.

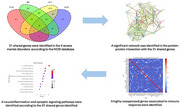

## 1 Introduction

Mental disorders exhibit significant heritability, with genetic factors accounting for 34%–77% of variability (Polderman et al. 2015). Schizophrenia (SZ), bipolar disorder (BD), and neurodevelopmental disorders (NDD) show higher heritability compared to major depressive disorder (MDD) and post‐traumatic stress disorder (PTSD) (Faraone et al. 2005, Lichtenstein et al. 2009, Mullins et al. 2021, Niemi et al. 2018, Schizophrenia Working Group of the Psychiatric Genomics Consortium 2014).

Genome‐wide association studies (GWAS) have revealed substantial genetic overlap among these conditions, with ∼75% of loci shared between at least two disorders (Caspi and Moffitt 2018, Grotzinger 2021). This genetic pleiotropy challenges traditional categorical diagnoses and supports the need for transdiagnostic approaches (Anttila et al. 2018, Bourque et al. 2024). The shared genetic architecture of these disorders often involves pathways linked to immune regulation, neurotransmitter signaling, and neuroplasticity (Romero et al. 2022, Schulze et al. 2014). For instance, altered dopamine and glutamate signaling, as well as immune dysregulation, have been implicated in SZ, BD, and MDD (Schulze et al. 2014). However, the lack of comprehensive analyses integrating multiple data sources hinders the identification of common biological mechanisms that may explain their overlapping genetic basis.

This study leverages large‐scale public genetic datasets to systematically explore the shared genetic architecture of MDD, BD, SZ, and PTSD. Using a systems biology approach, we analyzed intersecting genetic loci to identify key protein–protein interactions (PPIs) and enriched biological pathways. By focusing on common molecular mechanisms, this research aims to inform transdiagnostic therapeutic strategies and advance our understanding of the biological underpinnings of severe mental disorders.

## 2 Material and Methods

### 2.1 Selection of Psychiatry Conditions

Based on the meta‐analysis by Duncan et al. (2017) that identified genetic overlap among four severe mental illnesses—PTSD, MDD, BD, and SZ—we selected these disorders for our exploratory analysis.

### 2.2 Identification of Genes Related to MDD Versus BD Versus SZ Versus PTSD

We identified disorder‐related genes for MDD, BD, SZ, and PTSD using the NCBI Gene database, a curated and integrated resource within the National Center for Biotechnology Information (NCBI). The Gene database aggregates information from various sources, with a core foundation in the NCBI Reference Sequence (RefSeq) project, ensuring standardization and high‐quality annotations (Brown et al. 2015). For each disorder, we conducted independent keyword‐based queries using the official disorder MeSh term in the Gene database. We applied the following inclusion criteria to extract relevant genes: (1) statistical threshold: only genes reported in GWAS or related genetic studies with genome‐wide significance were included (*p* < 5 × 10^−8^); (2) functional annotation: genes had to be associated with the disorder through curated records supported by functional or expression‐based evidence; and (3) redundancy filter: duplicate entries, non‐human genes, and genes lacking clear phenotype association were excluded.

The output from each disorder‐specific query included information such as gene name, gene ID, description, chromosomal location, aliases, and associated Mendelian Inheritance in Man (MIM) numbers. These results were downloaded in tab‐delimited text format and subsequently converted into structured .xlsx files for further processing and analysis. To manage large gene counts, we prioritized genes based on the combination of statistical significance (*p* < 5 × 10^−8^), evidence of expression in brain tissue, via links to gene expression databases such as GTEx, and relevance in previously published psychiatric genomics literature.

### 2.3 Gene Intersection Between Severe Mental Disorders

We built a database with the identified and curated genes from the NCBI and their association with each disorder. Each column represents a group of genes according to the following assignment: group A: PTSD; group B: MDD; group C: BD; group D: SZ. We use the open‐software R to identify the intersection of gene data according to all combinations: PTSD versus MDD; MDD versus BD; PTSD versus BD; PTSD versus SZ; MDD versus SZ; BD versus SZ; PTSD versus MDD versus BD; PTSD versus MDD versus SZ; MDD versus BD versus SZ; PTSD versus BD versus SZ; PTSD versus MDD versus BD versus SZ.

### 2.4 Protein–Protein Interaction Network Analysis

The intersected genes associated with these disorders were analyzed using the STRING database v12.0 to predict PPIs. STRING integrates five main interaction sources: genomic context prediction, high‐throughput experiments, conserved coexpression patterns, automated text mining, and prior database knowledge. Using protein nomenclature from the intersection analyses, networks were created where nodes represent proteins and edges indicate functional associations. Two network types were analyzed: (1) edges by evidence, where line colors indicate interaction evidence type, and (2) edges by confidence, where line thickness reflects data support strength. A minimum interaction score of 0.4 (medium confidence) was applied, representing an approximate confidence level based on cumulative evidence (Szklarczyk et al. 2019).

Two network analyses were conducted: the first included all interaction sources, and the second excluded text mining, neighborhood, gene fusion, and co‐occurrence, retaining only experimental evidence, curated pathways, protein‐complex knowledge, and coexpression data. Markov Cluster Algorithm (MCL) analysis was performed using an inflation parameter of 4 to control clustering granularity (Enright 2002). This parameter ranging between 1.2 and 5.0 controls the granularity of the output clustering (The mcl manual n.d.). All protein‐interaction analyses were applied to the intersected outputs of MDD, BD, SZ, and PTSD.

### 2.5 Coexpression Analysis

Coexpression analysis based on RNA expression patterns and protein co‐regulation were provided by ProteomeHD. ProteomeHD is a dataset that cover 294 biological conditions using isotope‐labeling mass spectrometry and applying the machine learning algorithm TreeClust to reveal functional associations between co‐regulated human proteins from ProteomeHD (Kustatscher et al. 2019). STRING uses the results of the TreeClust Algorithm with a recalibrate and score using the KEGG benchmark (Szklarczyk et al. 2019).

### 2.6 Gene Set Enrichment Analysis

Gene set enrichment analysis (GSEA) is a statistical method for interpreting high‐throughput expression studies, using Gene Ontologies (GO terms) and expression data to uncover biological processes and pathways linked to specific phenotypes (Zito et al. 2021). The STRING database provided GSEA outputs, including GO terms for biological processes, molecular functions, cellular components, KEGG pathways, and tissue expression data. Analyses applied a false discovery rate (FDR) threshold of 0.05 using the Benjamini–Hochberg procedure for multiple comparisons.

The network output variables included two key counts: (1) the number of proteins within the network annotated with a specific term and (2) the total number of proteins (network and background) associated with that term. Enrichment strength quantified the magnitude of enrichment as the ratio between the observed proteins annotated with a term and the expected number in a random network of the same size (Szklarczyk et al. 2023).

## 3 Results

### 3.1 Gene Intersections

We analyzed genes associated with MDD, BD, SZ, and PTSD, as derived from the NCBI database: MDD (410 genes), BD (765 genes), SZ (1,893 genes), and PTSD (99 genes). Gene intersection analysis identified shared genes between disorders, including PTSD versus MDD (41), MDD versus BD (147), PTSD versus BD (47), PTSD versus SZ (63), MDD versus SZ (214), and BD versus SZ (459). Multigroup intersections included PTSD versus MDD versus BD (33), PTSD versus MDD versus SZ (36), MDD versus BD versus SZ (129), PTSD versus BD versus SZ (44), and all four disorders (31).

**FIGURE 1:**
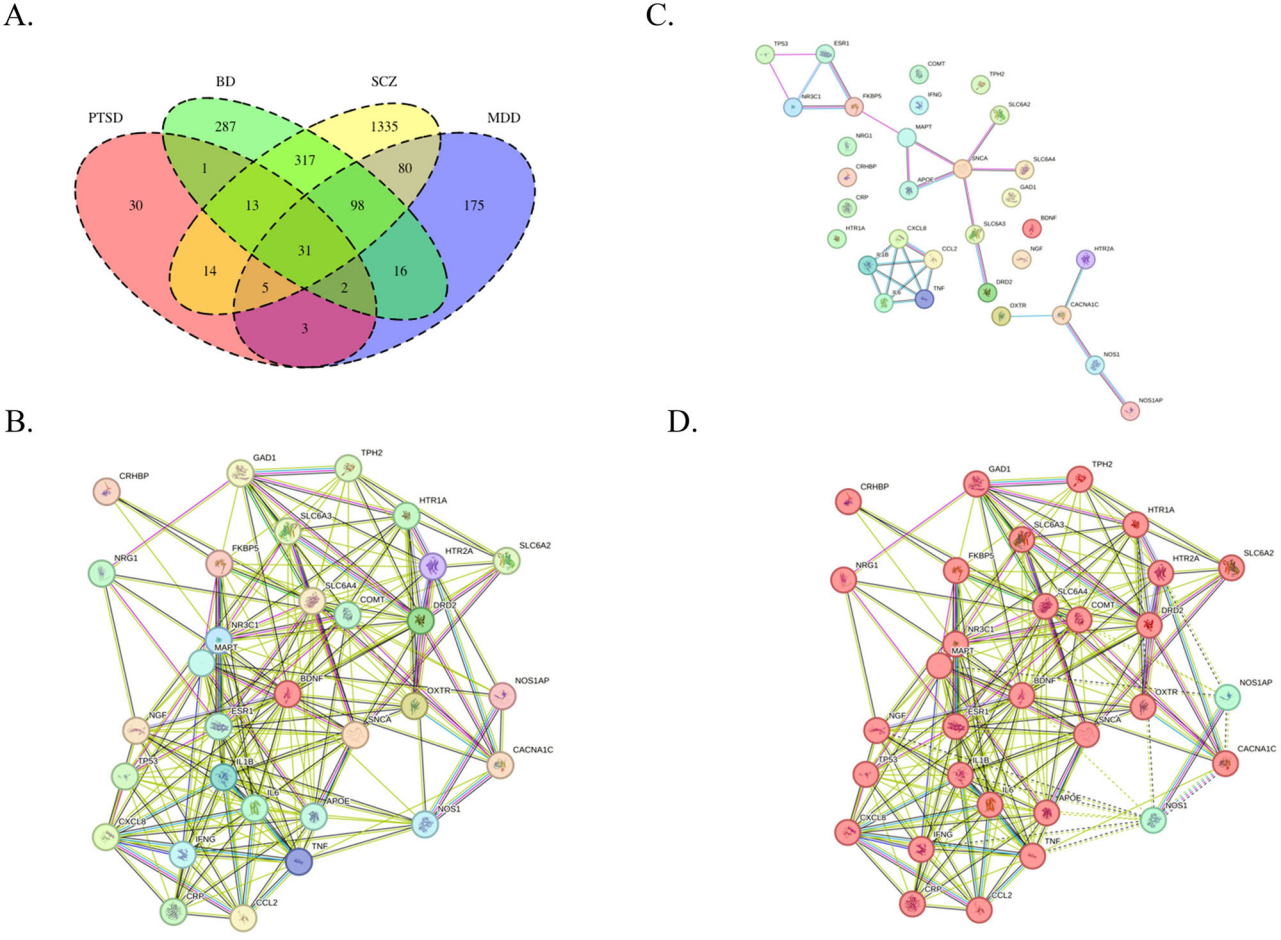
Protein–protein interaction network of MDD versus BD versus SZ versus PTSD (A). Venn diagram of MDD versus BD versus SZ versus PTSD (B). Protein–protein interaction network of MDD versus BD versus SZ versus PTSD (C). PPI network MDD versus BD versus SZ versus PTSD (including only database, co‐expression, experiments) (D). MCL algorithm graph from 31 genes intersected.

A Venn diagram was generated to visualize overlapping genes across PTSD, MDD, BD, and SZ (Figure 1A; Table 1).

**TABLE 1:** Description of 31 intersected genes of MDD versus BD versus SZ versus PTSD.

Gene	Function	REFERENCE
APOE	*Apolipoprotein E* encodes a major apoprotein of chylomicrons. Essential for catabolism of lipoproteins.	HGNC:HGNC:613
BDNF	*Brain‐Derived Neurotrophic Factor* encodes a member of the nerve growth factor family proteins. Plays a role in neuroplasticity.	HGNC:HGNC:1033
CACNA1C	*Calcium Voltage‐Gated Channel Subunit Alpha 1C* encodes a subunit of a calcium channel that mediates the influx of calcium into the cell.	HGNC:HGNC:1390
CCL2	*C‐C Motif Chemokine Ligand 2* is one of several cytokine genes. It has been implicated in the pathogenesis of diseases characterized by monocytic infiltrates, such as psoriasis, rheumatoid arthritis, and atherosclerosis.	HGNC:HGNC:10618
COMT	*Catechol‐O‐Methyltransferase* catalyzes the transfer of a methyl group from S‐adenosylmethionine to catecholamines, including the neurotransmitters dopamine, epinephrine, and norepinephrine.	HGNC:HGNC:2228
CRHBP	*Corticotropin Releasing Hormone Binding Protein*. Corticotropin‐releasing hormone is a potent stimulator of synthesis and secretion of preopiomelanocortin‐derived peptides.	HGNC:HGNC:2356
CRP	*C‐reactive protein* is a protein coding gene. Displays several functions associated with host defense.	HGNC:HGNC:2367
CXCL8	*C‐X‐C Motif Chemokine Ligand 8*. The protein encoded by this gene is a member of the CXC chemokine family and is a major mediator of the inflammatory response. The encoded protein is commonly referred to as interleukin‐8 (IL‐8).	HGNC:HGNC:6025
DRD2	*Dopamine Receptor D2* encodes the D2 subtype of the dopamine receptor. A missense mutation in this gene causes myoclonus dystonia; other mutations have been associated with schizophrenia.	HGNC:HGNC:3023
ESR1	*Estrogen Receptor 1* encodes an estrogen receptor and ligand‐activated transcription factor. The protein encoded by this gene regulates the transcription of many estrogen‐inducible genes that play a role in growth, metabolism, sexual development, gestation, and other reproductive functions and is expressed in many non‐reproductive tissues.	HGNC:HGNC:3467
FKBP5	*FKBP Prolyl Isomerase 5*. The protein encoded by this gene is a member of the immunophilin protein family, which plays a role in immunoregulation and basic cellular processes involving protein folding and trafficking.	HGNC:HGNC:3721
GAD1	*Glutamate Decarboxylase 1* encodes one of several forms of glutamic acid decarboxylase, identified as a major autoantigen in insulin‐dependent diabetes. The enzyme encoded is responsible for catalyzing the production of gamma‐aminobutyric acid from l‐glutamic acid.	HGNC:HGNC:4092
HTR1A	*5‐Hydroxytryptamine Receptor 1A* encodes a G protein‐coupled receptor for 5‐hydroxytryptamine (serotonin) and belongs to the 5‐hydroxytryptamine receptor subfamily. Serotonin has been implicated in a number of physiologic processes and pathologic conditions.	HGNC:HGNC:5286
HTR2A	*5‐Hydroxytryptamine Receptor 2A* encodes one of the receptors for serotonin, a neurotransmitter with many roles. Mutations in this gene are associated with susceptibility to schizophrenia and obsessive‐compulsive disorder and are also associated with response to the antidepressant citalopram in patients with major depressive disorder (MDD).	HGNC:HGNC:5293
IFNG	*Interferon Gamma* encodes a soluble cytokine that is a member of the type II interferon class, which plays crucial roles in antimicrobial, antiviral, and antitumor responses by activating effector immune cells and enhancing antigen presentation.	HGNC:HGNC:5438
IL1B	*Interleukin 1 Beta*. The protein encoded by this gene is a member of the interleukin 1 cytokine family. This cytokine is an important mediator of the inflammatory response and is involved in a variety of cellular activities, including cell proliferation, differentiation, and apoptosis.	HGNC:HGNC:5992
IL6	*Interleukin 6* encodes a cytokine that functions in inflammation and the maturation of B cells. The functioning of this gene is implicated in a wide variety of inflammation‐associated disease states, including susceptibility to diabetes mellitus and systemic juvenile rheumatoid arthritis.	HGNC:HGNC:6018
MAPT	*Microtubule Associated Protein Tau* encodes the microtubule‐associated protein tau and promotes microtubule assembly and stability and might be involved in the establishment and maintenance of neuronal polarity. Mutations have been associated with several neurodegenerative disorders such as Alzheimer's disease, Pick's disease, frontotemporal dementia, cortico‐basal degeneration, and progressive supranuclear palsy.	HGNC:HGNC:6893
NGF	*Nerve Growth Factor* encodes a secreted protein which homodimerizes and is incorporated into a larger complex. This protein has nerve growth‐stimulating activity and the complex is involved in the regulation of growth and the differentiation of sympathetic and certain sensory neurons.	HGNC:HGNC:7808
NOS1	*Nitric Oxide Synthase 1*. The protein encoded by this gene belongs to the family of nitric oxide synthases, which synthesize nitric. Nitric oxide is a reactive free radical, which acts as a biologic mediator in several processes, including neurotransmission, and antimicrobial and antitumoral activities. In the brain and peripheral nervous system, nitric oxide has been implicated in neurotoxicity associated with stroke and neurodegenerative diseases, neural regulation of smooth muscle, including peristalsis, and penile erection.	HGNC:HGNC:7872
NOS1AP	*Nitric Oxide Synthase 1 Adaptor Protein* encodes a cytosolic protein that binds to the signaling molecule, neuronal nitric oxide synthase (nNOS).	HGNC:HGNC:16859
NR3C1	*Nuclear Receptor Subfamily 3 Group C Member 1* encodes glucocorticoid receptor, which can function both as a transcription factor and as a regulator of other transcription factors. It is involved in inflammatory responses, cellular proliferation, and differentiation in target tissues.	HGNC:HGNC:7978
NRG1	*Neuregulin 1*. The protein encoded by this gene is a membrane glycoprotein that mediates cell–cell signaling and plays a critical role in the growth and development of multiple organ systems. Dysregulation of this gene has been linked to diseases such as cancer, schizophrenia, and bipolar disorder (BPD).	HGNC:HGNC:7997
OXTR	*Oxytocin Receptor*. The protein encoded by this gene belongs to the G‐protein coupled receptor family and acts as a receptor for oxytocin. Its activity is mediated by G proteins which activate a phosphatidylinositol‐calcium second messenger system. The oxytocin–oxytocin receptor system plays an important role in the uterus during parturition.	HGNC:HGNC:8529
SLC6A2	*Solute Carrier Family 6 Member 2* encodes a member of the sodium: neurotransmitter symporter family. This member is a multi‐pass membrane protein, which is responsible for reuptake of norepinephrine into presynaptic nerve terminals and is a regulator of norepinephrine homeostasis.	HGNC:HGNC:11048
SLC6A3	*Solute Carrier Family 6 Member 3* encodes a dopamine transporter which is a member of the sodium‐ and chloride‐dependent neurotransmitter transporter family.	HGNC:HGNC:11049
SLC6A4	*Solute Carrier Family 6 Member 4* encodes an integral membrane protein that transports the neurotransmitter serotonin from synaptic spaces into presynaptic neurons. The encoded protein terminates the action of serotonin and recycles it in a sodium‐dependent manner. It is a target of psychomotor stimulants, such as amphetamines and cocaine, and is a member of the sodium: neurotransmitter symporter family.	HGNC:HGNC:11050
SNCA	*Synuclein Alpha* is a member of the synuclein family, which also includes beta‐synuclein and gamma‐synuclein. It may serve to integrate presynaptic signaling and membrane trafficking. SNCA peptides are a major component of amyloid plaques in the brains of patients with Alzheimer's disease.	HGNC:HGNC:11138
TNF	*Tumor Necrosis Factor* encodes a multifunctional proinflammatory cytokine that belongs to the tumor necrosis factor (TNF) superfamily. This cytokine has been implicated in a variety of diseases, including autoimmune diseases, insulin resistance, psoriasis, rheumatoid arthritis ankylosing spondylitis, tuberculosis, autosomal dominant polycystic kidney disease, and cancer.	HGNC:HGNC:11892
TP53	*Tumor Protein P53* encodes a tumor suppressor protein. The encoded protein responds to diverse cellular stresses to regulate expression of target genes, thereby inducing cell cycle arrest, apoptosis, senescence, DNA repair, or changes in metabolism.	HGNC:HGNC:11998
TPH2	*Tryptophan Hydroxylase 2* encodes a member of the pterin‐dependent aromatic acid hydroxylase family. The encoded protein catalyzes the first and rate limiting step in the biosynthesis of serotonin. Mutations in this gene may be associated with psychiatric diseases such as bipolar affective disorder and major depression.	HGNC:HGNC:20692

### 3.2 Protein–Protein Interaction and Network Characteristics

The intersection of MDD, BD, SZ, and PTSD yielded 31 genes (Figure 1A). The initial PPI network contained 31 nodes and 187 edges, with an average node degree of 12.1, an average clustering coefficient of 0.689, and a highly significant PPI enrichment *p* value (<1.0 × 10^−16^). A confidence analysis using a minimum interaction score of 0.400 was also generated (Figure 1B).

A second network excluded text mining, neighborhood, gene fusion, and co‐occurrence, retaining only experimental evidence, curated pathways, and co‐expression data. This network included 31 edges, an average node degree of 1.74, an average clustering coefficient of 0.487, and a PPI enrichment *p* value of 1.48 × 10^−11^ (Figure 1C).

The Markov cluster algorithm (MCA) analysis produced two clusters. Cluster 1 included 29 genes with 197 edges, an average node degree of 13.6, an average clustering coefficient of 0.75, and a PPI enrichment *p* value <1.0 × 10^−16^. Cluster 2 consisted of two nodes and one edge, with an average node degree of 1, a clustering coefficient of 1, and a PPI enrichment p value of 0.011 (Figure 1D).

### 3.3 Functional Enrichment Analysis of the Intersected Genes

**FIGURE 2:**
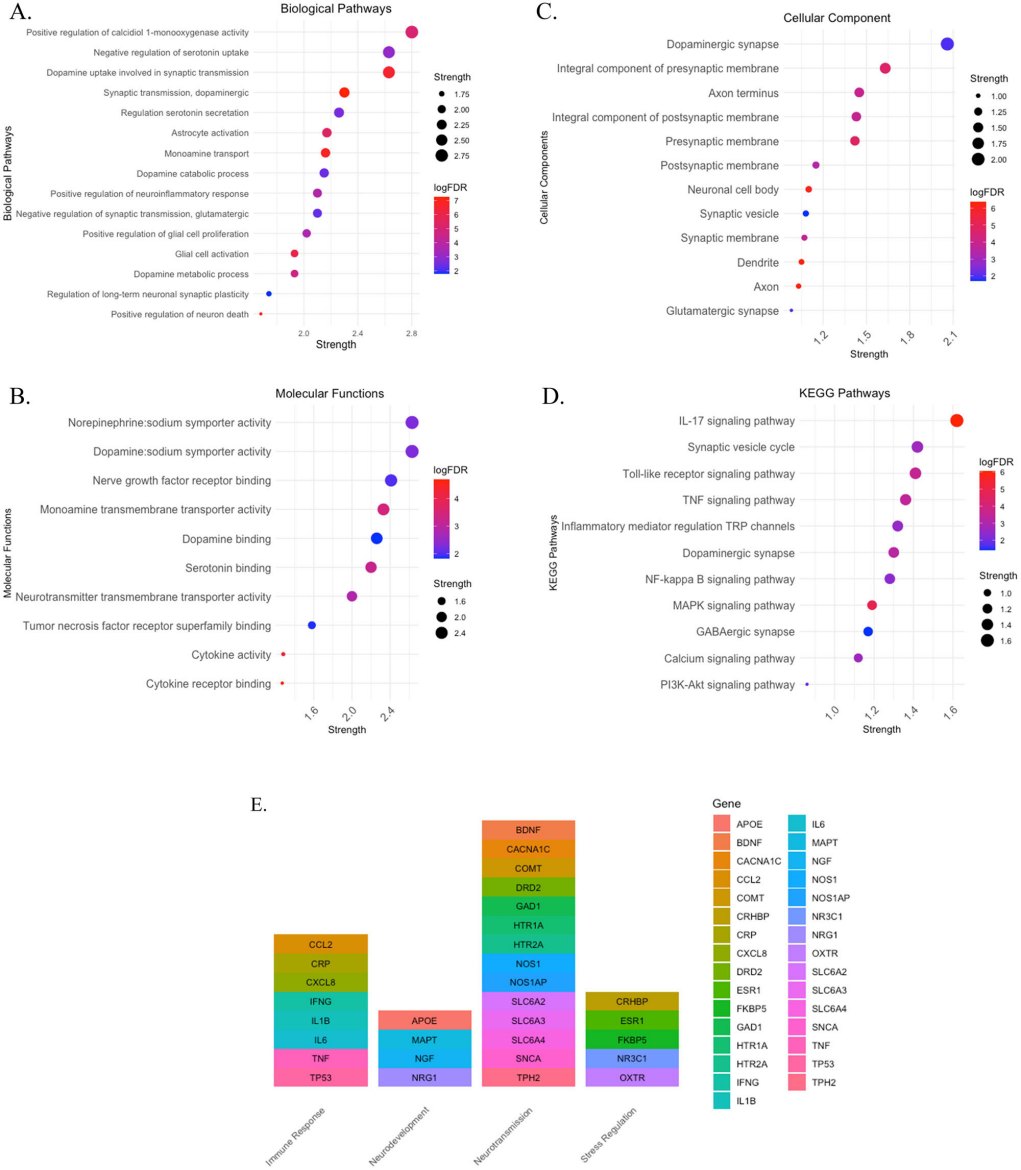
Functional enrichment analysis of the 31 genes selected by MDD versus BD versus SZ versus PTSD (A). Biological pathways (B). Molecular functions (C). Cellular component (D). KEGG pathways (E). Genes grouped by categories.

In the functional enrichment analysis, we identified several significantly enriched biological pathways, molecular functions, cellular components, and tissue expression within the selected gene set. For biological pathways, positive regulation of calcidiol1‐monooxygenase activity showed the largest enrichment connectivity (*S* = 2.98, *p* = 7.6 × 10^−6^). Neurotransmitter involved in synaptic transmission also shows significant enrichments connectivity such as dopamine (*S* = 2.3, *p* = 5.63 × 10^−08^) and serotonin secretation (*S* = 2.26, *p* = 0.0029; see Figure 2A). In the molecular functions, symporter activity in monoamines such as norepinephrine sodium symporter (*S* = 2.63, *p* = 0.0053), and dopamine sodium symporter (*S* = 2.63, *p* = 0.0053) also shows significant enrichments connectivity. Binding proteins receptor such as dopamine, serotonin, and cytokines also show significant enrichment, see Figure 2B. Cellular components identified in the network with significant larges enrichment are implied in dopaminergic synapse (*S* = 2.06, *p* = 0.0111), integrate component of presynaptic membrane (*S* = 1.63, *p* = 1.93 × 10^−05^), and integrate component of postsynaptic membrane (*S* = 1.43, *p* = 0.00013). KEGG pathways analysis demonstrates a large enrichment for IL‐17 signaling pathway (*S* = 1.62, *p* = 9.02 × 10^−07^), synaptic vesicle cycle (*S* = 1.42, *p* = 0.0020), tumor necrosis factor (TNF) signaling pathway (*S* = 1.36, *p* = 0.0003), among others. See Figure 2D and Tables S1–S4.

### 3.4 Coexpression Analysis of the Intersected Genes

**FIGURE 3:**
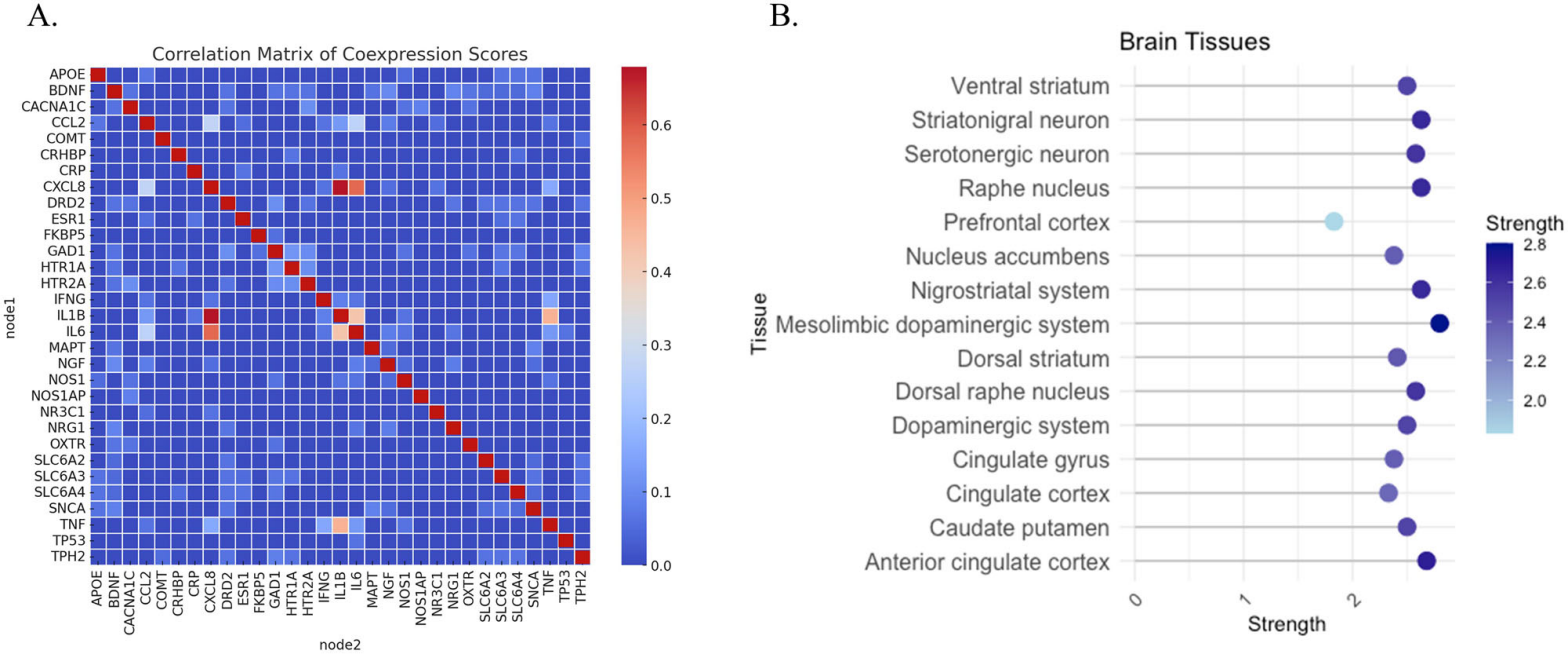
(A) Coexpression scores based on RNA expression patterns of 31 genes selected from STRING database and ProteomeHD and (B) tissue expression of the 31 genes selected in brain tissues.

A co‐expression analysis was conducted to predict functional associations among genes. The resulting heatmap displays co‐expression scores derived from RNA expression patterns scores across the 31 selected genes (see Figure 3A). A table with the total co‐expression scores is in Table S6.

### 3.5 Tissue‐Specific Expression

A significant largest enrichment of the 31 intersected genes expression was identified in brain tissue. Mesolimbic dopaminergic system (*S* = 2.8, *p* = 0.00032) and anterior cingulate cortex (*S* = 2.68, *p* = 7.42 × 10^−06^) exhibited the largest enrichment. Other areas such as nigrostriatal system (*S* = 2.63, *p* = 0.0005), raphe nucleus (*S* = 2.63, *p* = 2.10 × 10^−07^), and caudate putamen (*S* = 2.5, *p* = 1.37 × 10^−05^) also show significant enrichment connectivity. See Figure 3B and Table S5.

## 4 Discussion

This study identified 31 intersected genes among MDD, BD, SZ, and PTSD from a public database resulted from experimental studies. The PPI was strongly significant between the 31 genes. The above was evidence at the confluence of interleukin signaling, TNF signaling, inflammatory mediator regulation TRP channels pathways, among others. Additionally, neurotransmitter regulation, synaptic transmission, stress regulation, and neurodevelopmental and glial activation were also identified as biological pathways. A higher co‐expression was identified between inflammatory and immune response pathways genes. The enrichment of the 31 intersected genes in brain regions such as the mesolimbic dopaminergic system and anterior cingulate cortex suggests a shared genetic impact on neural circuits involved in reward, emotion regulation, and cognitive control.

For the discussion of our results and previous published research, the intersected genes identified can be grouped in four categories: (i) immune response and inflammation, (ii) neurotransmission and synaptic function, (iii) neuroendocrine and stress regulation, and (iv) neurodevelopmental and structural integrity.

### 4.1 Immune Response and Inflammation

The immune system plays a central role in the pathophysiology of severe mental disorders. Our analysis identified IL‐1β and IL‐8 as shared genetic markers across MDD, BD, SZ, and PTSD, reinforcing the significance of neuroimmune dysregulation as a transdiagnostic mechanism. IL‐1β, a prototypical pro‐inflammatory cytokine, modulates neuronal proliferation, synaptic plasticity, and apoptosis (Mendiola and Cardona 2018). Its elevated peripheral levels and functional polymorphisms are consistently associated with MDD susceptibility and treatment response (Bhattacharya et al. 2016, Tsai 2017, Zhang et al. 2023). Moreover, IL‐1β levels are increased in BD and SZ and contribute to neuroinflammatory cascades involved in memory, fear processing, and emotional dysregulation in PTSD (Bhattacharya et al. 2016, Brunoni et al. 2020, Jones and Thomsen 2013, Réus et al. 2015).

IL‐8 showed coexpression with IL‐1β in our dataset. Though IL‐8 findings in MDD are heterogeneous, its elevation has been observed during BD depressive episodes and is linked to illness course in SZ (Eyre et al. 2016, Tsai 2021). These patterns suggest IL‐8 may function as a state‐dependent marker of inflammatory activation, particularly relevant in mood disorders. In PTSD, limited but emerging evidence implicates IL‐8 in glial activation and stress‐induced neuroinflammation (Jones and Thomsen 2013).

Our results align with large‐scale cross‐disorder meta‐analyses, where 30 of 44 inflammatory markers were dysregulated in at least one psychiatric disorder (Yuan et al. 2019). C‐reactive protein (CRP) and IL‐6 were elevated in MDD, SZ, and PTSD, while CCL2 was particularly increased in MDD (Yuan et al. 2019). Notably, IL‐1β distinguishes MDD from BD, being elevated in the former but reduced in the latter (Yuan et al. 2019). Mendelian randomization studies support a causal role for IL‐6 in MDD and SZ, further affirming the mechanistic involvement of chronic low‐grade inflammation in disease onset and progression (Perry et al. 2021, Zhang et al. 2023).

### 4.2 Neurotransmission and Synaptic Function

Disrupted neurotransmitter signaling, especially within the dopaminergic and serotonergic systems, emerged as a key feature in our analysis. The dopamine receptor gene DRD2, consistently implicated in GWAS of SZ and BD, was central in our PPI network (Cabana‐Domínguez et al. 2022). In SZ, DRD2 polymorphisms are associated with increased receptor expression and D2S/D2L splice variant imbalances, contributing to psychotic symptoms (Kaalund et al. 2014). In BD, increased D2L expression aligns with reward hypersensitivity during manic episodes (Kaalund et al. 2014). In PTSD and MDD, DRD2 variants also affect mood regulation and stress responsivity (Zhang et al. 2023).

The dopamine transporter gene SLC6A3 (DAT1) regulates synaptic dopamine clearance (Reith et al. 2022). Our findings link this gene to both BD and MDD, supported by evidence showing reduced DAT levels in MDD and polymorphic variations such as Glu602Gly that increase BD risk (Reith et al. 2022). However, preclinical studies showing protective effects of DAT knockdown on anxiety suggest complex, possibly compensatory dynamics between gene expression and disease states (Bahi and Dreyer 2019, Perona et al. 2008, Pizzagalli et al. 2019).

The serotonergic system was also broadly implicated. 5‐HTR1A polymorphisms such as rs6295 have been associated with MDD, BD, and SZ. Reduced 5‐HT1A receptor binding in the medial prefrontal cortex and hippocampus contributes to MDD pathophysiology, while genetic variation predicts SSRI treatment response (Kishi et al. 2013, Lin et al. 2015, Ramesh et al. 2022, Savitz et al. 2009). 5‐HTR2A (e.g., rs6313) and SLC6A4 (5‐HTTLPR) variants further support serotonergic dysregulation, especially in MDD and SZ (Lin et al. 2015, Petit et al. 2014). Notably, individuals with the l/l genotype of 5‐HTTLPR show better SSRI responses and reduced SZ risk, linking synaptic serotonin transport to clinical outcomes (Lin et al. 2015, Petit et al. 2014, Ramesh et al. 2022).

### 4.3 Neuroendocrine and Stress Regulation

Genes regulating hypothalamic–pituitary–adrenal (HPA) axis activity, particularly FKBP5 and NR3C1, were identified as shared across MDD, PTSD, and BD (Menke et al. 2013, Szczepankiewicz et al. 2014, Q. Wang et al. 2018, Zannas et al. 2016). FKBP5 regulates glucocorticoid receptor sensitivity and is implicated in stress reactivity, especially in individuals with early‐life trauma (Q. Wang et al. 2018). Its functional variants such as rs1360780 are associated with increased psychiatric vulnerability in gene–environment interaction models (Hawn et al. 2019). NR3C1, encoding the glucocorticoid receptor, modulates transcriptional responses to stress hormones and is dysregulated in both MDD and PTSD (Sheerin et al. 2020).

OXTR, encoding the oxytocin receptor, also emerged as relevant in SZ and BD. Dysfunctions in the oxytocin system have been associated with impaired social cognition and increased cardiometabolic comorbidity, supporting recent findings from UK Biobank studies that link OXTR polymorphisms with social‐affective deficits and metabolic syndrome in psychiatric populations (Winterton et al. 2021).

### 4.4 Neurodevelopmental and Structural Integrity

Our findings also support the convergence of psychiatric risk on neurodevelopmental and neurostructural genes. APOE, traditionally associated with neurodegenerative disorders, was identified as a cross‐disorder gene due to its role in lipid metabolism, synaptic stability, and neuroinflammation (Li et al. 2024). The ε4 allele increases risk for late‐life depression, worsens cognitive outcomes in BD (Bellivier et al. 1997, De Souza et al. 2010, Feng et al. 2015, Kerr et al. 2016), and contributes to SZ‐related brain abnormalities via disrupted cholesterol regulation and interactions with reelin (Digney et al. 2005, Gibbons 2011, Vila‐Rodriguez 2011).

NGF and NRG1 play pivotal roles in neuronal survival and synaptic plasticity. Reduced NGF expression has been found in MDD and SZ, while NRG1 modulates HPA axis activity and microglial function (Ceci et al. 2021, Rao et al. 2017, W. Wang et al. 2020, Xu et al. 2020). Its expression in the medial prefrontal cortex is critical for stress resilience, and deficiencies are associated with depression‐like phenotypes. NRG1 dysregulation has also been linked to altered dendritic morphology and neuroinflammatory responses in SZ, with implications for BD and PTSD (Chohan et al. 2014, Clarke et al. 2019, Taylor et al. 2011).

NOS1 and NOS1AP, which regulate nitric oxide production and NMDA receptor activity, were also common to our intersected gene set. These genes modulate glutamatergic transmission and are involved in synaptic plasticity, neurovascular regulation, and stress‐related memory formation. Variants in NOS1 have been linked to SZ and MDD, with peripheral NO levels correlating with depression severity (Freudenberg et al. 2015, Kudlow et al. 2016, Mcneill et al. 2022, Weber et al. 2014). In PTSD, genetic variation in NOS1AP is associated with greater symptom burden, particularly among combat‐exposed individuals (Bruenig et al. 2017, Fronza et al. 2023, Lawford et al. 2013).

### 4.5 Tissue‐Specific Findings

A key finding from our tissue enrichment analysis is the significant overrepresentation of the 31 intersected genes in brain regions implicated in reward, motivation, and affect regulation. The mesolimbic dopaminergic system showed the strongest enrichment (*S* = 2.8, *p* = 0.00032), underscoring its role in anhedonia, motivational deficits, and mood dysregulation (Der‐Avakian and Markou 2012, Nestler and Carlezon 2006). In MDD, blunted dopaminergic signaling in the ventral tegmental area–nucleus accumbens pathway contributes to reduced reward anticipation and pleasure (Cui et al. 2024, Marx et al. 2023). Preclinical models confirm decreased dopamine release and altered excitatory–inhibitory balance in this circuit (Belujon and Grace 2017, Yadid and Friedman 2008).

The anterior cingulate cortex (ACC), another highly enriched region (*S* = 2.68, *p* = 7.42 × 10^−6^), is implicated in cognitive‐emotional integration and self‐referential processing (Stevens et al. 2011). In MDD, disrupted connectivity in ACC subregions correlates with symptom severity and treatment resistance (Barthas et al. 2017, Davey et al. 2012, Peng et al. 2021, Rolls 2019, Zhou et al. 2024). Transcriptomic analyses in BD and SZ show downregulation of immune and synaptic genes in the ACC, suggesting a convergent mechanism involving neuroinflammation and synaptic dysfunction (Adams and David 2007, Nelson et al. 2015, Zandi et al. 2022). PTSD studies also show reduced immune gene expression in this region, consistent with stress‐induced transcriptomic reprogramming (Jaffe et al. 2022).

## 5 Conclusions

This study highlights a significant shared genetic architecture among major psychiatric disorders such as MDD, BD, SZ, and PTSD, through the identification of 31 intersected genes enriched in pathways related to immune response and neuroinflammation, neurotransmitter regulation and synaptic plasticity, neuroendocrine and stress function, finally neurodevelopmental and structural integrity. Central among these were cytokine signaling (e.g., IL‐1β, IL‐8) and dopaminergic transmission (DRD2, SLC6A3), underscoring the intricate crosstalk between the immune system and brain function in the pathophysiology of severe mental illness.

GWAS further support a substantial, though non‐identical, genetic overlap across these disorders. Shared loci such as CACNA1C, BDNF, and COMT exhibit pleiotropic effects on emotion regulation, cognition, and stress responsivity. Genetic correlation analyses report coefficients as high as *r* = 0.83 (Romero et al. 2022), while polygenic risk score (PRS) studies show that individuals with a high genetic burden for one disorder (e.g., BD) frequently carry overlapping risk variants for MDD and SZ (Andlauer et al. 2021). Notably, multi‐trait GWAS and PRS × environment interaction models (e.g., with childhood trauma in PTSD) highlight the complexity and dynamic nature of shared vulnerability (Duncan et al. 2017, Ratanatharathorn et al. 2021).

By integrating data from GWAS, PPI networks, and brain‐region‐specific enrichment analyses, this study emphasizes the value of a systems biology approach to psychiatric genomics. The findings challenge traditional categorical diagnostic boundaries by revealing convergent biological substrates underlying distinct clinical phenotypes.

This transdiagnostic framework opens new avenues for the development of integrative and personalized therapeutic strategies that target common molecular pathways across diagnostic categories. Future research should aim to validate these genetic networks in larger and more diverse populations, examine their translational relevance to symptom dimensions and disease trajectories, and investigate their interaction with environmental risk factors to inform precision psychiatry.

## Author Contributions

**Hernan F. Guillen‐burgos**: conceptualization, investigation, funding acquisition, writing – original draft, methodology, validation, visualization, writing – review and editing, formal analysis, project administration, data curation. **Valentina Vanegas**: methodology, writing – review and editing, data curation. **Isabela Gonzalez**: formal analysis, data curation. **Ana M. Caicedo**: formal analysis, data curation. **Valentina Arango**: visualization, data curation. **Juan F. Galvez‐Florez**: supervision, writing – original draft. **Juan‐Manuel Anaya**: investigation, writing – review and editing, writing – original draft. **Roger S. Mcintyre**: writing – review and editing, writing – original draft, conceptualization, supervision. **Wilson Terán**: conceptualization, validation, methodology, formal analysis, data curation, supervision.

## Ethics Statement

This research did not require IRB/EC approval. This is a without risk study because the information obtained is available in public databases and does not involve human subjects or animals.

## Conflicts of Interest

H.F.G.B. has received research grant support from the Ministry of Science, Technology, and Innovation (MinCiencias) in Colombia, UKRI in the United Kingdom; and speaker fees from Abbott, GSK, Roche, Pfizer, Synergy R&D. V.V., A.C., I.G., V.A., J.F.G.F., J.M.A., and W.T. declare no conflicts of interest to report. R.S.M. has received research grant support from CIHR/GACD/National Natural Science Foundation of China (NSFC) and the Milken Institute; speaker/consultation fees from Lundbeck, Janssen, Alkermes, Neumora Therapeutics, Boehringer Ingelheim, Sage, Biogen, Mitsubishi Tanabe, Purdue, Pfizer, Otsuka, Takeda, Neurocrine, Sunovion, Bausch Health, Axsome, Novo Nordisk, Kris, Sanofi, Eisai, Intra‐Cellular, NewBridge Pharmaceuticals, Viatris, Abbvie, and Atai Life Sciences. He also is the CEO of Braxia Scientific Corp.

## Peer Review

The peer review history for this article is available at https://publons.com/publon/10.1002/brb3.70742.

## Supporting information




**Supplementary Tables**: brb370742‐sup‐0001‐tablesS1‐S6.xlsx

## Data Availability

Data are available upon request from the corresponding authors.
